# Primary versus Nonprimary Cytomegalovirus Infection during Pregnancy, Israel

**DOI:** 10.3201/eid1311.061289

**Published:** 2007-11

**Authors:** Galia Rahav, Rinat Gabbay, Asher Ornoy, Svetlana Shechtman, Judith Arnon, Orna Diav-Citrin

**Affiliations:** * Hadassah Medical Center–Hebrew University School of Medicine, Jerusalem, Israel; † Israel Ministry of Health, Jerusalem, Israel; 1 Current affiliation: Sheba Medical Center, Tel-Hashomer, Israel

**Keywords:** Cytomegalovirus, pregnancy, primary infection, nonprimary infection, dispatch

## Abstract

We examined prospectively the outcome of primary and nonprimary maternal cytomegalovirus (CMV) infection during pregnancy among 88 and 120 women, respectively. The risk for vertical transmission was 1.83× higher for primary infection than for nonprimary infection. Nonetheless, congenital CMV disease was diagnosed in both infection groups at similar rates.

Cytomegalovirus (CMV) infection is the most frequent congenital infection and a common cause of deafness and intellectual impairment, affecting 0.5%–2.5% of all live births (*1*–*3*). Intrauterine infection occurs in 40% of primary maternal infections, with delivery of 10% to 15% symptomatic newborns and late neurologic sequelae in 10% of those asymptomatic at birth (*1*).

Although preexisting maternal immunity reduces maternal-fetal transmission, the severity of congenital CMV disease is similar following primary or nonprimary infection (*4*–*7*). Yet, several reports found increased vertical transmission after nonprimary CMV infection (*4*–*9*). Therefore, our objective was to examine the outcome of primary and nonprimary maternal CMV infections during pregnancy.

## The Study

Institutional Ethics Committee approval was obtained. Women with positive CMV immunoglobulin (Ig) M (n = 208), referred for risk for CMV infection between January 1998 and December 2001, were enrolled in this prospective cohort observational study. Clinical and pregnancy-related information was obtained. Serum CMV IgG and IgM were measured by enzyme immunoassay and CMV-IgM immunofluorescence assay (*10*). IgG avidities <25% indicated recent infection (*10*).

Ultrasonographic examinations were performed between the 15th and 21st weeks of pregnancy. The reference method for prenatal diagnosis of CMV, requiring combined viral isolation and positive CMV PCR from amniotic fluid after gestational week 21 or 7 weeks after maternal symptoms (*3*,*11*), was applied for all amniocenteses. Amniotic fluid samples were inoculated onto MRC5 monolayers for CMV isolation (*10*), and DNA was amplified by PCR (*10*,*12*). Parents made decisions regarding amniocentesis and the fate of pregnancy after medical, and sometimes rabbinical, consultations. Elective terminations of pregnancy (ETOP) required external committee approval. Available aborted fetuses were examined for CMV-induced histopathologic changes. Immediately after birth, neonatal urine and anti–CMV IgM were examined. Subsequently, the newborns underwent cerebral ultrasound and auditory and ophthalmologic assessment.

Primary infection was defined as the occurrence of anti–CMV IgG seroconversion during pregnancy (*1*). Women who were seropositive for anti–CMV IgM and anti–CMV IgG when first evaluated during pregnancy and with IgG avidity >35% were considered to have nonprimary infection (*12*). The latter were divided into those with preconception evidence of anti–CMV IgG and negative anti–CMV IgM (group 1) and those without prior tests for CMV (group 2). Vertical transmission was declared if the amniotic fluid contained CMV virus or DNA, if pathologic features of CMV disease existed in the aborted fetus, or if neonatal IgM or urine cultures were positive for CMV.

Analysis of variance and the Kruskal-Wallis or Mann-Whitney tests were used. Frequencies were compared by χ^2^ or Fisher exact tests. Relative risk was calculated with Epi Info 2000 software (available from www.cdc.gov/epiinfo).

Of the 208 enrolled women, 88 (42.3%) had primary CMV infection; 120 (57.7%) had nonprimary CMV infection, 36 (17.3%) from group 1 and 84 (40.4%) from group 2. The mothers’ ages were similar in both groups. The median gestational age upon referral was 15 weeks (9.5–19.0 weeks), and the median number of pregnancies was 3 (range 1–10). CMV serologic testing was part of the routine gynecologic examination in 127 (61.0%) of the women: 35 (39.8%) after primary infection and 92 (76.6%) in the nonprimary infection group (p<0.001). Clinical signs of CMV infection triggered 52 (25%) of the tests, while patient anxiety induced the rest. Clinical CMV symptoms were more common with primary than with nonprimary infections (53 [60.2%], and 44 [36.6%], respectively, p = 0.002).

Pregnancies with primary infection had significantly fewer live births than those with nonprimary infection (Table 1). Primary infections in the first 20 gestational weeks resulted in 46.5% live births, 46.5% ETOP, and 7% miscarriages, while pregnancies with such infections after week 23 were 100% full term (p = 0.004).

**Table 1:** Outcome of pregnancies by type of CMV infection*

Outcome of pregnancy	Primary infection (%)	Nonprimary infection (%)	Total	p value
Live birth	51 (58.0)	97 (80.8)	148	<0.001
ETOP*	30 (34.0)	21 (17.5)	51	0.006
Miscarriage†	7 (8.0)	2 (1.7)	9	0.038
Total	88	120	208	

*CMV, cytomegalovirus; ETOP, elective termination of pregnancy. Thirty ETOP were performed during the first trimester, 21 between the 21st and 23rd weeks of pregnancy. †Mean gestational age of miscarried fetuses was 7 wk.

The following analysis included 169 women (excluding 39 with miscarriages or ETOP before week 21). Of them, 100 had amniocentesis, with most in the nonprimary infection group 2, 62.7% (52/83), and the rest similarly distributed between nonprimary group 1, 42.9% (15/35), and primary infection, 40.7% (33/81). Approximately half of the amniocenteses provided evidence of fetal CMV infection, both in primary infection (16/33) and nonprimary infection group 1 (7/15), but only in 17.3% (9/52) of group 2 (p<0.001). Vertical transmission was determined from the amniotic fluid by culture (n = 12) or PCR (n = 30), abortus pathology (n = 6), or positive neonatal IgM (n = 2) or urine cultures (n = 13). Vertical transmission rates were 35.8% (24/76), 30.0% (9/30), and 15.3% (11/72) in the primary and nonprimary infection groups 1 and 2, respectively (p = 0.017). The relative risk for vertical transmission in primary infection was 1.83 (95% confidence interval 1.1–3.03, p = 0.019) versus nonprimary infection.

All liveborn babies had similar natal characteristics regardless of the maternal infection group. Four newborns and 7 aborted fetuses (6.5%, 11/169) had congenital CMV disease (Table 2), 6.0% (4/67) after primary CMV infection and 6.9% (7/102) after nonprimary infection, with 13.3% (4/30) in group 1 and 4.2% (3/72) in group 2 (p = 0.26). Three of the 4 mentally retarded neonates were born after nonprimary infection. CMV infection was not detected in newborns of mothers with negative prenatal diagnostic tests (n = 68).

**Table 2:** Characteristics of aborted fetuses and neonates with congenital CMV disease*

Case no.	Maternal infection	Maternal symptoms	Week of infection	US	AF	Outcome, wk	Natal/abortion status	Follow-up, 2 y
1	Primary	Flu	<20	IUGR microcephaly	+	Live born, 38	Congenital disease	Sensorineural hearing loss, PMR
2	Primary	None	<18	Hyperechoic bowel	ND	Abortion, 19	Postmortem: disseminated CMV	
3	Primary	Fever	7	Hyperechoic bowel	+	Abortion, 23	Postmortem: disseminated CMV	
4	Primary	Fever	12	None	+	Abortion, 24	Postmortem: disseminated CMV	
5	Nonprimary (group 1)	None	Unclear	IUGR	ND	Live born, 38	Brain calcifications	PMR
6	Nonprimary (group 1)	Flu	22	Tricuspid regurgitation	ND	Abortion, 33	Postmortem: disseminated CMV	
7	Nonprimary (group 1)	Fever	20	IUGR pericardial fluid, brain calcifications	+	Abortion, 30	Postmortem: disseminated CMV	
8	Nonprimary (group 1)	Flu	<20	None	+	Live born, 40	Congenital disease	PMR
9	Nonprimary (group 2)	Fever	12	ND	+	Abortion, 24	Postmortem: disseminated CMV	
10	Nonprimary (group 2)	Flu	4	ND	ND	Live born, 38	Congenital disease	Sensorineural hearing loss, PMR
11	Nonprimary (group 2)	None	14	Liver calcifications	+	Abortion, 24	Postmortem: disseminated CMV	

*CMV, cytomegalovirus; US, ultrasonograph; AF, amniotic fluid; IUGR, intrauterine growth retardation; +, positive according to PCR, culture, or both; ND, not done; PMR, psychomotor retardation.

## Conclusions

To our knowledge, this is the first cohort in which the natural history of nonprimary CMV infection was evaluated prospectively in the mother from pregnancy to its conclusion, in contrast to published studies that determined it from established neonatal infection and retrospective assessment of the mothers’ serologic test results (*2*,*4*–*8*,*13*). Most Israeli physicians obtain CMV serologic test results in pregnancy in response to women’s demands and as a precaution, but the results, mainly of nonprimary infection, are confusing. Our findings suggest that nonprimary infection is also dangerous. While vertical transmission after primary CMV infection was similar to the reported 30% to 40% (*1*,*3*,*11*), after nonprimary infection it was 19.6%, much higher than the published 2.2% (*1*–*3*,*13*). Furthermore, of the 11 cases of congenital CMV disease, 7 were associated with nonprimary maternal infection.

The high rate of vertical transmission in the nonprimary infection group attests to the numerous amniocenteses performed in Israel to exclude fetal infection. The procedure is considered safe because neonatal loss rates in those undergoing it and not undergoing it are identical (*14*), and the risks of undergoing the procedure do not outweigh the risks for congenital CMV infection. In the nonprimary group, 67 (55.8%) had amniocentesis, but in the primary infection group, only 33 (37.5%) underwent the procedure, which reflects the increased early ETOP rate with primary infection.

Traditionally, vertical transmission after nonprimary infection was established by isolating CMV from neonatal saliva or urine, or the presence of neonatal IgM (*4*–*8*). We, however, determined transmission also from CMV in the amniotic fluid, and the observed difference between amniotic CMV and neonatal CMV disease suggests fetal virus elimination between amniocentesis and birth, probably by preexisting maternal CMV-specific antibodies. The high transmission rate could have resulted also from reinfection by CMV strains different from the primary strain (*6*), further enhanced by increased virulence of present-day strains.

The high proportion (>40%) of religiously observant women accounts for the reluctance of some to terminate pregnancy without ultrasonographic and amniocentesis evidence of fetal infection and also for the increased early ETOP (permitted by Jewish law only before the second trimester). We are aware that data were obtained for only 169/199 (84.9%) of fetuses or newborns and that only 31/51 (60.8%) of the aborted fetuses were available for examination, but omissions were random. Possible referral selection bias and the low number of affected women discourage definitive conclusions. Nevertheless, both primary and nonprimary CMV infection during pregnancy are clearly important causes of congenital disease.
